# A Pre-experimental Study to Assess the Effectiveness of Planned Teaching Program on Knowledge and Expressed Practices Regarding Selected Obstetrical Emergencies Among Staff Nurses in Selected Hospitals of Shimla District, Himachal Pradesh

**DOI:** 10.7759/cureus.29811

**Published:** 2022-10-01

**Authors:** Bhavna Verma, Manoj Kumar, Swati Swati, Kamla Tanwar, Shashi Kiran

**Affiliations:** 1 Obstetrics and Gynecology, All India Institute of Medical Sciences, Deoghar, Deoghar, IND; 2 Pediatrics, All India Institute of Medical Sciences, Deoghar, Deoghar, IND; 3 Obstetrics and Gynecological Nursing, Sister Nivedita Government Nursing College, Shimla, IND; 4 Child Health Nursing, Indira Gandhi Medical College, Shimla, IND

**Keywords:** midwives, midwifery, knowledge, planned teaching program, obstetrical emergencies, staff nurses, expressed practices

## Abstract

Background and objective

Good health and well-being occupy the third position among 17 sustainable development goals designed by the United Nations. The key to reducing maternal and newborn morbidity and mortality is competent and skilled birth attendance. The objectives of this study were to assess and compare the pre-test and post-test knowledge and expressed practices regarding selected obstetrical emergencies among staff nurses; to develop and determine the effectiveness of planned teaching programs on selected obstetrical emergencies among staff nurses; and to find out the correlation between knowledge and expressed practices regarding selected obstetrical emergencies.

Materials and methods

A pre-experimental study was conducted for a period of one month in 2019 among 60 staff nurses in selected hospitals through a validated tool/questionnaire, which was piloted on six staff nurses prior to starting the study. Data were collected using a structured knowledge questionnaire and expressed practices checklist.

Results

Of note, 70% of participants had General Nursing and Midwifery (GNM) as a professional qualification. The majority (51.7%) had one to five years of work experience; 46.7% of staff nurses had good knowledge in the pre-test assessment and 95% had good knowledge in the post-test evaluation. Significantly, 80% showed good expressed practices in the pre-test and 96.7% revealed good expressed practices in the post-test regarding selected obstetrical emergencies. In the pre-test, there was a significant association between the sociodemographic variables (age and work experience) with expressed practices, while that was not the case with post-test expressed practices. No significant association was found between pre- and post-test knowledge and selected demographic variables. There was a significant difference between pre-test and post-test knowledge and expressed practices score (mean pre- and post-test knowledge score: 18.82 vs. 25.43, p<0.001; mean pre- and post-test expressed practices score: 14.43 vs. 16.30, p<0.001).

Conclusion

Based on our findings, the planned teaching program is effective in improving the knowledge and expressed practices of staff nurses regarding selected obstetrical emergencies.

## Introduction

The quality of healthcare is determined by the availability of resources, the actual provision of care, and the patient’s level of satisfaction. Nurses represent the largest workforce in the field, and they play an important role as primary healthcare providers. Their preparedness for obstetrical emergencies is of paramount importance in reducing maternal and neonatal mortality and morbidity. Conducting regular training programs for the staff nurses can help to improve the expected level of care.

The key to reducing maternal and newborn morbidity and mortality is competent skilled birth attendance. Worldwide, from 2015 to 2021, approximately 84% of births were assisted by skilled health professionals, which included medical doctors, nurses, and midwives. This number was 77% from 2008 to 2014. Although many children are still dying, progress has been observed in under-five and neonatal mortality [1]. Ensuring healthy lives and promoting well-being at all ages has to be a priority for all countries and communities. Unless and until the quality of care is improved and standardized, the desired impact cannot be achieved by just increasing the number of health facilities.

Midwifery has not received the importance it deserves and is often neglected, although a series of four papers in the Lancet and evidence in 2014 suggested that more than 80% of maternal and newborn deaths including stillbirths could be averted by strengthening midwifery services across the world [2]. Midwifery skills can effectively provide many maternal and newborn health interventions like basic emergency obstetrics and neonatal Care (BEmONC; i.e., assisted delivery, removal of retained products, manual removal of the placenta, administration of oxytocic drugs, antibiotics, and anticonvulsants, and neonatal resuscitation) [3].

India's maternal mortality ratio (MMR) was 103 in 2017-2019 as compared to 113 in 2016-2018 according to the special bulletin on MMR released by the Registrar General of India on March 14, 2022; 130 or more maternal deaths per one lakh live births indicates very high MMR [4]. Himachal Pradesh's MMR in 2015-2016 was 63. Thirteen sick newborn care units (SNCU), 49 newborn stabilizing units (NBSU), and 120 newborn care corners (NBCC) are functioning in the state [5].

The healthcare system at the community level needs preparedness for obstetric emergencies. Ensuring quality care to everybody irrespective of socioeconomic class can only be attained if constant training is given to all the healthcare workers to handle various emergency situations. Nurses, midwives, and paramedical staff should know the outline and flowchart of the management of obstetrics and other concerned emergencies. The WHO also advocates the same and hence midwifery toolkit and education modules have been available on the WHO website for a couple of decades along with the Quality of Care Midwifery Network Webinar since March 2021 [6]. 

A structured teaching program can be a part of training the nursing team to enhance their knowledge, attitude, skill, and competence. Developing procedural skills, and simulations using models and mannequins can be readily incorporated into training programs with potential benefits for teaching infrequently performed or more difficult procedures.

The null hypothesis of the present study assumes that there is no difference between pre- and post-test knowledge and expressed practice scores of staff nurses on implementing planned teaching programs in selected obstetrical emergencies.

## Materials and methods

A prospective study was undertaken in May 2019 at the Kamla Nehru State Hospital for Mother and Child, Shimla, and Deen Dayal Upadhyay Zonal Hospital, Shimla in India. It included 60 staff nurses (30 from each hospital) as study subjects.

The tool/questionnaire consisted of three sections: section A included demographic variables such as age, professional qualifications, work experience, current working area, and source of the previous information regarding obstetrical emergencies; section B entailed a structured questionnaire to assess knowledge (30 items) with multiple-choice questions with four options and one correct answer. Out of 30 items, 15 were related to preeclampsia and eclampsia, four were about placenta previa, nine were related to abruptio placenta, and the remaining were related to general obstetrics; section C consisted of a structured checklist to assess the expressed practices (expressed practice checklist), which had only two responses: "yes" or "no". A score of 1 was given for the correct response while it was 0 for the wrong response. Unattempted questions were marked 0. For the knowledge domain, a total score ranging from 0 to 10 was assessed as poor knowledge, 11 to 20 as average, and 21 to 30 as good. For expressed practices, a total score ranging from 0 to 10 was assessed as a poor level and 11 to 20 as a good level.

To ensure content validity, the tool was submitted to nine different experts. The r-value for self-structured knowledge was 0.93 and for expressed practices, it was again 0.93. So, the questionnaire was deemed reliable. Piloting of the study was done in April 2019 on six staff nurses at the Kamla Nehru State Hospital for Mother and Child, Shimla. 

Data collection was done in three steps. In the first step, a pre-test was taken using a structured questionnaire and expressed practice checklist. The planned teaching program was given (as small group teaching) on the same day as the second step. After seven days, post-test data were collected using the same tool from the same participants as the third and last step of data collection. Data were entered into a Microsoft Excel sheet 2010.

Sociodemographic variables were described/expressed in frequencies and percentages, mean, mean percentage, and standard deviation (SD); inferential measures "paired t-test" was used to assess the effectiveness of the planned teaching program. The coefficient of correlation was used to find out the correlation between knowledge and expressed practices. The chi-square test was used to determine the association of knowledge and expressed practice with sociodemographic variables. Data analysis was done using SPSS Statistics 2018 version (IBM Corp., Armonk, NY).

## Results

Table 1 depicts the demographic variables of the study subjects (n=60). The majority (48.3%, 29) were of 21-30 years, 38.3% (23) were in the age group of 31-40 years, 11.7% (seven) were in the 41-50 years age group, and 1.7% (one) was >50 years old. With respect to professional qualifications, the majority (70%, 42) was General Nursing and Midwifery (GNM), 20% (12) were B.Sc. Nursing, 10% (six) were post-B.Sc. Nursing, but none were M.Sc. Nursing. Of note, 8.3% (five) had <1 year of work experience, 51.7%, i.e., the majority (31), had a work experience ranging from one to five years, 31.7% (19) had a work experience of 6-10 years, and 8.3% (five) had >10 years of work experience. For the majority (58.3%, 35), the source of previous information was continuing/in-service education, followed by other sources (25%, 15); the curriculum was the source of information in 11.7% (seven) and 5% (three) got information from workshops. The current working area for the majority of staff nurses was the labor room (16.75%, 10), followed by 15% (nine) each for NICU, OT, and the antenatal ward.

**Table 1:** Demographic characteristics of respondents MCH: maternal and child health; NICU: neonatal intensive care unit; OT: operation theater; SNCU: sick newborn care unit

Variables		Frequency (f) (n=60)	Percentage (%)
Age (years)	21-30	29	48.3
	31-40	23	38.3
	41-50	7	11.7
	>50	1	1.7
Academic qualifications	10+2/intermediate	43	71.7
	Graduate	16	26.7
	Post-graduate	1	1.7
	Any other	0	0
Professional qualifications	GNM (General Nursing and Midwifery)	42	70
	Basic B.Sc. Nursing	12	20
	Post-basic B.Sc. Nursing	6	10
	M.Sc. Nursing	0	0
Work experience (years)	<1	5	8.3
	1-5	31	51.7
	6-10	19	31.7
	>10	5	8.3
Source of previous information	Curriculum	7	11.7
	Workshop	3	5
	Continuing/in-service education	35	58.3
	Any other medium	15	25
Current working area	Antenatal ward	9	15
	Blood bank	2	3.3
	Gynecology ward	3	5
	Labor room	10	16.7
	Pre-/early-labor ward	5	8.3
	MCH counseling zone	4	6.7
	NICU	9	15
	OT	9	15
	SNCU	2	3.3
	Postnatal ward	1	1.7
	Special ward	3	5
	Surgical ward	3	5

Table 2 shows the pre-test knowledge score regarding obstetrical emergencies. Scores were good (21-30) for 46.7% of participants (28), average (11-20) for 45% of participants (27), and poor (0-10) for 8.3% of participants (five). The mean score was 18.82, and the median was 19 (standard deviation: 6.113); the minimum score was 7 and the maximum was 29.

**Table 2:** Pre-test knowledge score SD: standard deviation

Pre-test knowledge score level	Values (n=60)
Poor (0-10), n (%)	5 (8.3)
Average (11-20), n (%)	27 (45)
Good (21-30), n (%)	28 (46.7)
Mean	18.82
Median	19
SD	6.113
Range	22
Maximum	29
Minimum	7
Mean %	62.72

Table 3 shows the post-test score. Of note, 95% (57) showed good knowledge, 5% (three) had average knowledge, and none (0%) showed poor knowledge. The mean score was 25.43, the median was 26, the standard deviation was 3.259, the maximum score obtained was 33, and the minimum was 17.

**Table 3:** Post-test knowledge score SD: standard deviation

Post-test knowledge score level	Values (n=60)
Poor (0-10), n (%)	0 (0)
Average (11-20), n (%)	3 (5)
Good (21-30), n (%)	57 (95)
Mean	25.43
Median	26
SD	3.259
Range	16
Maximum	33
Minimum	17
Mean %	84.78%

Table 4 depicts the pre-test expressed practice score. Among the respondents, 12 (20%) showed poor practices (0-10) and 48 (80%) showed good practices. The score for pre-test expressed practices showed a mean of 14.43, a median of 15, and a standard deviation of 3.212; 12 was the maximum obtained score and 8 was the minimum, and the mean percentage score was 72.17%.

**Table 4:** Pre-test expressed practice score SD: standard deviation

Pre-test expressed practice score level	Values (n=60)
Poor (0-10), n (%)	12 (20)
Good (21-30), n (%)	48 (80)
Mean	14.43
Median	15
SD	3.212
Range	12
Maximum	20
Minimum	8
Mean %	72.17%

Table 5 shows the post-test expressed practice scores. Of note, 96.7% (58) had a good score and 3.3% (two) showed a poor score. The mean score for post-test expressed practices was 16.3, the median was 17, the standard deviation was 2.331, the range was 8, and the maximum and minimum obtained scores were 19 and 11 respectively, while the mean percentage was 81.5%.

**Table 5:** Post-test expressed practices scores SD: standard deviation

Post-test expressed practice score level	Values (n=60)
Poor (0-10), n (%)	2 (3.3)
Good (21-30), n (%)	58 (96.7)
Mean	16.3
Median	17
SD	2.331
Range	8
Maximum	19
Minimum	11
Mean %	81.50%

A comparison of pre-test and post-test scores after the planned teaching program was performed. Table 6 shows the comparison of descriptive statistics. The mean post-test knowledge score (25.43) was higher than the mean pre-test knowledge score (18.82). The pre-test and post-test knowledge scores of staff nurses regarding selected obstetrical emergencies were calculated by paired t-test, and the ‘t’ value of 9.817 shows the significant association between pre-test and post-test knowledge. The mean post-test expressed practices score (16.30) was higher than the pre-test expressed practices score (14.43). The pre-test and post-test expressed practices score of staff nurses regarding selected obstetrical emergencies was calculated by paired t-test, and the ‘t’ value of 4.333 shows the significant association between pre-test and post-test expressed practices.

**Table 6:** Comparison of descriptive statistics SD: standard deviation

Paired t-test		Mean	SD	Mean %	Mean difference	Paired t-test	P-value	Table value at 0.05
Knowledge	Pre	18.82	6.113	62.72	6.617	9.817	<0.001	2.00
	Post	25.43	3.259	84.78				
Expressed practices	Pre	14.43	3.212	72.17	1.867	4.333	<0.001	2.00
	Post	16.30	2.331	81.50				

**Figure 1:**
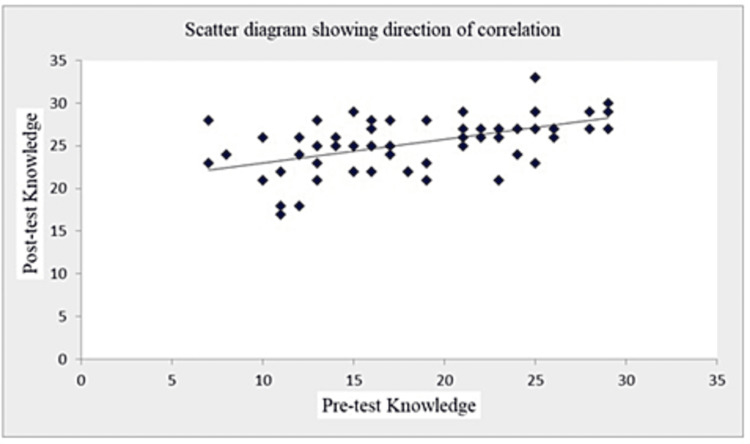
Shows the significant correlation between pre-test knowledge and post-test knowledge scores

**Figure 2:**
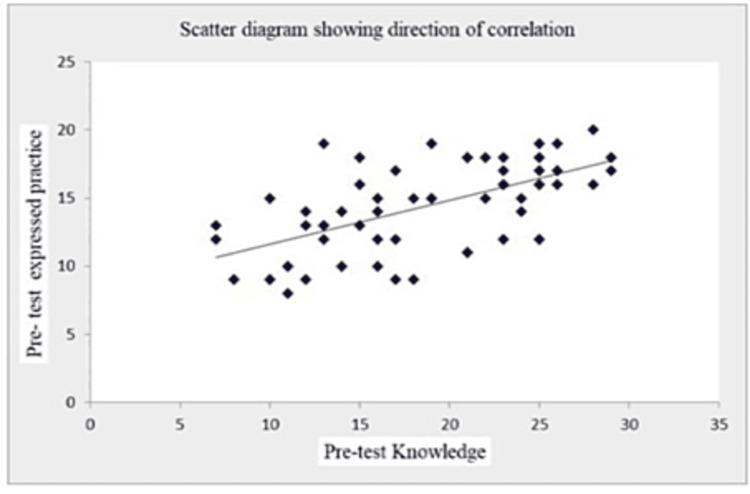
Shows the significant correlation between pre-test knowledge and pre-test expressed practices scores

**Figure 3:**
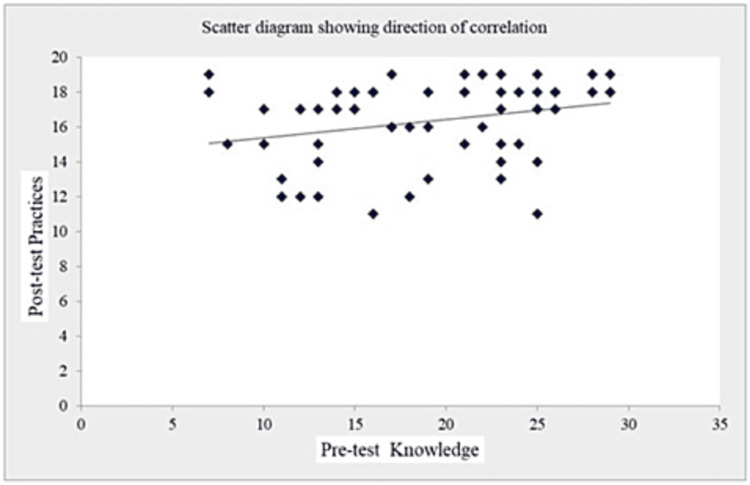
Shows the significant correlation between pre-test knowledge and post-test expressed practices scores

**Figure 4:**
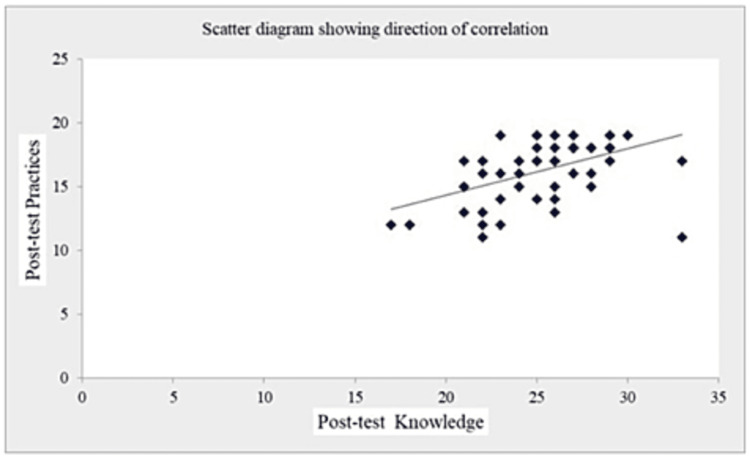
Shows the significant correlation between post-test knowledge and post-test expressed practices scores

**Figure 5:**
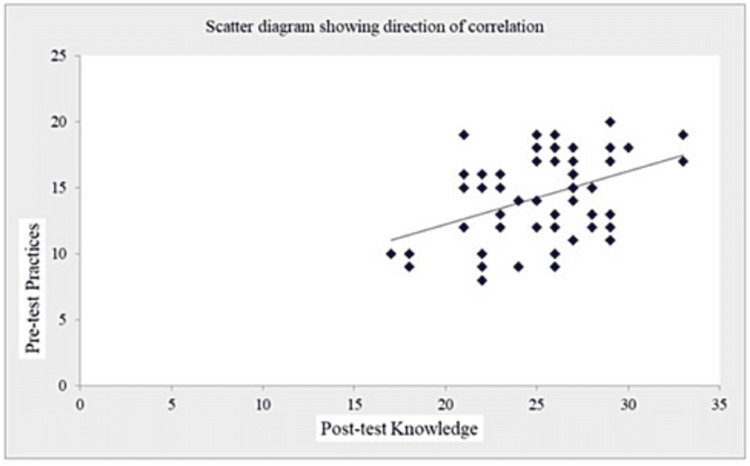
Shows the highly significant correlation between post-test knowledge and pre-test expressed practices scores

The coefficient of correlation (r-value) in Table 7 and Figures 1-5 (scatter diagrams) are significant for pre-test and post-test knowledge and expressed practices, which shows that the planned teaching program was quite helpful in improving knowledge and level of expressed practice. The r-value for pre-test knowledge versus post-test knowledge was 0.520 (p=0.001); for pre-test knowledge versus pre-test practices, it was 0.613 (p=0.001); for pre-test knowledge versus post-test practices, it was 0.276 (p=0.033); for post-test knowledge versus pre-test practices, it was 0.408 (p=0.001); for post-test knowledge versus post-test practices, it was 0.516 (p=0.001); and for pre-test practices versus post-test practices, it was 0.308 (p=0.017).

**Table 7:** Correlation between knowledge and expressed practices regarding obstetrical emergencies among staff nurses *Significant at p≤0.05. **Moderately significant at p≤0.01. ***Highly significant at p≤0.001

Pair 1	VS	Pair 2	r-value	P-value	Result
Pre-test knowledge	Vs	Post-test knowledge	0.520***	0.001	Significant
Pre-test knowledge	Vs	Pre-test practices	0.613***	0.001	Significant
Pre-test knowledge	Vs	Post-test practices	0.276*	0.033	Significant
Post-test knowledge	Vs	Pre-test practices	0.408***	0.001	Significant
Post-test knowledge	Vs	Post-test practices	0.516***	0.001	Significant
Pre-test practices	Vs	Post-test practices	0.308**	0.017	Significant

## Discussion

Improvement in the quality of healthcare services is only possible when the healthcare delivery system has technically competent health professionals who are able to provide Reproductive Maternal and Newborn Child Health and Adolescent (RMNCH+A) services. To ensure proficiency with regard to both technical skills and knowledge, a key intervention that is required is capacity building [7]. Capacity building can be achieved by planned teaching programs involving the relevant domains of knowledge and/or skills. In light of this, a study was done on 60 nurses working at government hospitals in obstetrics and gynecology in Shimla, India in May 2019, which involved a planned teaching program.

Our study included as many participants as possible with GNM qualifications (70%). Saifi et al. also conducted a similar descriptive evaluative study on 30 participants at the National Skills Training Center, Delhi. Most of the participants had Auxiliary Nursing Midwifery (ANM) qualifications (33.33%) in their study [8]. Kabi et al. conducted a prospective analytical study in 2020 for over two weeks on the coronavirus disease 2019 (COVID-19) airway training program involving 80 participants, which included registered nurses, technicians, and residents from anesthesiology, emergency medicine, trauma surgery, internal medicine, critical care medicine, pulmonary medicine, pediatrics, and otolaryngology departments. The objective was to assess the effectiveness of a simulation-based training program in improving knowledge and the concept of teamwork; 36.3% of the subjects were nurses/technicians [9].

A planned teaching program on the attitude of nurses with regard to patient rights was conducted in two multi-specialty teaching hospitals in Karnataka, India by D'Souza et al. with 200 nurses in the year 2017. The small group teaching method was used to train nurses in groups of 8-10. The post-test assessment was done through the same attitude questionnaire one week after the training program. The majority had one to three years of work experience [10]. The work experience of the majority of the participants (51.7%) in our study was one to five years, which is similar to the study by Gulista et al. [8] where 53.33% had 0-5 years of work experience.

In our study, 46.7%, 45%, and 8.3% had good (score: 21-30), average (score: 11-20), and poor (score: 0-10) pre-test knowledge, respectively. Regarding the pre-test, 20% and 80% of participants had inadequate (<68%) and adequate (≥68%) knowledge, respectively in the study by Saifi et al. [8]. In the study by Kabi et al., a feedback assessment was done, which showed that 58% and 52% of participants did agree that the module had a high impact on their knowledge and competence, respectively [9]. In the study by D'Souza et al., before training, 65% and 54% had favorable attitudes toward patient rights in hospital 1 and hospital 2, respectively. After training, 76% and 84% had the most favorable attitude toward patient rights in hospital 1 and hospital 2, respectively [10].

The mean post-test knowledge score (25.43) and mean post-test expressed practices score (16.30) were higher than their pre-test counterparts (pre-test knowledge score: 18.82 and pre-test practices score: 14.43). Paired t-tests and ‘t’ values for pre- and post-test of knowledge and expressed practices showed that the planned teaching program on obstetrical emergencies led to a significant and positive change in their knowledge and expressed practices. The coefficient of correlation between knowledge and expressed practice scores of training on MCH held at NSTC - “Daksh” is 0.46, showing a positive correlation between knowledge and expressed practice [8].

In the post-test, there was no statistically significant association between expressed practices with demographic variables (age, academic qualification, professional qualification, work experience, source of previous information, and current working area). The calculated chi-square values were less than the table value at the ≤0.05 level of significance in our study.

There was a significant difference between the post-test knowledge scores on the last day of training (K1) and knowledge scores assessed during the study (K2), which infers that the participants need more refresher training, in order to retain their knowledge and skills [8]. Motamed-Jahromi et al. (2011) suggested that more advocacy was needed by Iranian nurses (sample size: 385) through educational programs and support from responsible employers [11].

A Lives Saved Tool modeling study was done in 2019-20 to estimate the number of deaths that would be averted by 2035 if coverage of health interventions that can be delivered by professional midwives were scaled up in 88 countries which accounted for the majority of the world's maternal and neonatal deaths and stillbirths. A scale-up of midwife-delivered interventions like a 25% increase in coverage every five years from 2020 to 2035 in such countries would result in 41% fewer maternal deaths, 26% fewer stillbirths, and 39% fewer neonatal deaths relative to a scenario of no change in coverage. There are numerous barriers to enabling and supporting midwives in low-middle-income countries (LMICs): inadequate numbers of qualified midwives and unequal distribution, poor transport services, the high cost of accessing care, scarcity of supplies and equipment, improper education and regulation, and, in some countries, a lack of trust among the public due to previous experiences of disrespectful care. For midwives, barriers to providing high-quality care include social factors (e.g., gender inequality and exposure to violence), professional factors (e.g., gender issues, absence of midwives in policy dialogue, low recognition of midwifery skills by other professions, restrictions on practice, poor education, and scarcity of supplies and equipment), and economic factors (e.g., low or irregular salaries and poor housing and transport infrastructure) [12].

During the period spanning 2012-2014, knowledge and skills were evaluated among 5,939 healthcare providers before and after three to five days of "skills and drills" training in emergency obstetric and newborn care (EmOC&NC) in seven sub-Saharan African countries (Ghana, Kenya, Malawi, Nigeria, Sierra Leone, Tanzania, and Zimbabwe) and two Asian countries (Bangladesh and Pakistan). Standardized assessments were done using multiple choice questions and objective structured clinical examination (OSCE). Change in knowledge and skills was measured as well as the Improvement Ratio (IR) by cadre and by country [13]. Significant improvements were seen in knowledge and skills for each cadre of healthcare providers and for each country (p<0.05). The mean IR was 56% for doctors, 50% for mid-level staff and nurse-midwives, and 38% for nursing aides. This study concluded that additional support and training were required for monitoring progress in labor by the use of the partograph as a tool [13].

Limitations 

Our study has a few limitations. Primarily, our sample size (60) was small. Also, no attempt was made to do a follow-up study to measure the retention of knowledge among the samples. In addition, only selected obstetrical emergencies such as eclampsia, preeclampsia, placenta previa, and abruptio placentae were considered for the study. Another major limitation was that the study was limited to staff nurses only. Finally, only two hospitals were included in the study.

Strengths

Although the study was a small-scale analysis, it showed that planned teaching programs are simple interventions that would help increase the level of knowledge and standard of practice among staff nurses, especially in the field of obstetrical emergencies. In fact, the Lives Saved Tool modeling study [12] has shown that even a modest scale-up of coverage of midwife-delivered interventions (10% increase in coverage every five years from 2020 to 2035) would result in 22% fewer maternal deaths by 2035 (10 fewer per million), 14% fewer stillbirths (50 fewer per million), and 23% fewer neonatal deaths (100 fewer per million).

## Conclusions

Hospitals, clinics, and the entire healthcare system are supposed to fulfill the standard criteria laid down by the various approval bodies. Nurses and paramedical workers are the backbones of patient care not only in hospitals but also at community levels. Knowledge, attitude, and practice are three key components, each of which is of paramount importance in managing any emergency and routine patient care. The practice involves skill and competence.

Women's and children's health should be considered a priority. So, to manage obstetrical emergencies, expertise is expected in at least certain specified conditions. Hence, employers should ensure that nursing staff needs to undergo continuous training by attending planned teaching programs (theories and practical/simulation) periodically. A planned teaching program with pre-test assessment followed by post-test evaluation for the nursing staff is a cost-effective approach to improving maternal and newborn health outcomes.

## Appendices

Tool to Assess the Knowledge and Expressed Practices Regarding Obstetrical Emergencies Among Staff Nurses

Self-Structured Questionnaire

Date:                                                                                          Code No:

It has three sections:

Section A includes the sociodemographic profile.

Section B includes a structured questionnaire to assess the knowledge regarding certain obstetrical emergencies.

Section C includes a practice assessment checklist to assess the expressed practices regarding obstetrical emergencies.

Select the most suitable options and put a tick mark.

Information obtained from you will be kept confidential.

Section A

Sociodemographic Profile

1. Age (in years):

a) 21-30 years                                                                                                                 

b) 31-40 years                                                                                       

c) 41-50 years                                                                                       

d) > 50 years                                                                                         

 2. Academic qualifications                                                                                            

 a) 10+2                                                                                                

 b) Graduate                                                                                           

 c) Post Graduate

 d) Any other, specify……………………………                                                                                                                                          

 3. Professional qualifications:

 a) G.N.M                                                                                                                           

 b) Basic B.Sc. Nursing                                                                        

 c) Post Basic B.Sc. Nursing                                                                        

 d) M.Sc. Nursing                                                                                  

 4. Work experience:

 a) < 1 year                                                                                                                                                                                                     

 b) 1-5 years                                                                                          

 c) 6-10 years                                                                                        

 d) > 10 years                                                                                        

 5. Source of previous information:

 a) Curriculum                                                                                     

 b) Workshop                                                                                                                                               

 c) Continuing/in-service Education                                                        

 d) Any other medium, specify……………………..

 6. Current working area, specify ……………………….

Section B

Structured Questionnaire to Assess the Knowledge Regarding Obstetrical Emergencies Among Staff Nurses.

1. Obstetrical emergencies can occur

a) During pregnancy, labour and birth.

b) Only at labour and birth.

c) During pregnancy and birth.

d) Only at labour.

2. ‘Flashing lights’ is a symptom of :

a) Placenta previa

b) Abruptio placenta

c) Pre-eclampsia

d)  Hemorrhagic shock

3. In pre-eclampsia, vision is regained within:

a) 2-4 weeks

b) 1-2 weeks

c) 6-8 weeks

d) 4-6 weeks

4. Bio-chemical marker of pre-eclampsia is:

a) Hemoglobin

b) Serum uric acid

c) Albumin:Creatinine ratio

d) Beta HCG

5. Prevalence of eclamptic seizures are :

a) 2-3%

b) 10-15%

c) 20%

d) 30-40%

6. Sign of impending eclampsia is:

a) Epigastric pain

b) Sharp rise in B.P.

c) Back pain

d) Increase proteinuria

7. Choice of I/V fluid in patient with eclampsia is:

a) Ringer’s lactate

b) Normal saline 0.35%

c) Dextrose 5%

d) Dextrose Normal Saline

8. The principles behind the management of eclampsia are:

a) Maintain airway, breathing and circulation

b) Prevention of maternal injury and safe delivery

c) Arrest convulsions

d) All of the following

9. Convulsion in eclampsia is due to :

a) Shock

b) Cerebral anoxia due to arterial spasm

c) Hypocalcaemia

d) Hypovolemia

10. In eclamptic seizure, mouth gag should be removed after:

a) Clonic phase is over

b) Premonitory phase is over

c) Tonic phase is over

d) Tonic-clonic phase is over

11.  Loading dose of injection Magnesium Sulphate is:

a) 4gm I/V followed by 10gm I/M in each buttock.

b) 10gm I/V followed by 2 gm I/M in each buttock.

c) 15gm I/V followed by 6 gm I/M in each buttock.

d) 2gm I/V followed by 4 gm I/M in each buttock.

12. Maintenance dose of Inj. Magnesium sulphate is:

a) 10gm I.M. followed by 5gm I.M. every 4 hourly

b) 5gm I.M. followed by 2.5gm I.M. every 4 hourly

c) 8gm I.M. followed by 4gm I.M. every 2 hourly

d) 12gm I.M. followed by 6gm I.M. every 6 hourly

13.  Contraindication of magnesium sulfate is:

a) Diabetes

b) Hypercoagulation

c) Myasthenia gravis

d) Peptic ulcer

14.  Magnesium sulfate treatment is continued for:

a) 48-72 hours after last seizure

b) 12-24 hours after last seizure

c) 28-32 hours after last seizure

d) 8-12 hours after last seizure

15. Magnesium sulfate treatment is successful if:

a) BP is reduced to baseline

b) Seizure does not occur

c) Dieresis reduces fluid retention

d) Deep tendon reflex becomes hypotonic

16. Eclampsia has worst prognosis in:

a) Antenatal period

b) Post-natal period

c) Next pregnancy

d) Intrapartum period

17.  Placenta previa means:

a) Partial or total covering over the lower uterine segment by the placenta

b) Bleeding occurring due to premature separation of normally situated placenta

c) Abnormal formation of the placenta

d) Nausea and vomiting during pregnancy

18. Classical features of bleeding in placenta previa are:

a) Sudden onset, painful, apparently causeless and recurrent

b) Gradual onset, painful, apparently causeless and isolated

c) Sudden onset, painless, apparently causeless and recurrent

d) Gradual onset, painless, apparently causeless and isolated

19. High-risk factors in placenta previa is/are:

a) Multiparity and increased maternal age (> 35 years)

b) Nulligravida

c) History of normal vaginal delivery

d) Primigravida

20. Vaginal delivery is possible, when placenta is:

a) Clearly 2-3 cm away from the internal os

b) Clearly 1-1.5cm away from the internal os

c) Vaginal delivery cannot be performed in placenta previa

d) Completely covering the internal os

21. Steroid therapy is  indicated when duration of pregnancy is :

a) > 34 weeks

b) < 34 weeks

c) < 28 weeks

d) > 28 weeks

22. Abruptio placentae is also known as :

a) Accidental hemorrhage

b) Premature separation of placenta

c) Placental abruption

d) All of the above

23. Etiological factors of abruptio placentae is/are :

a) Excessive uptake of folic acid

b) Excessive uptake of carbohydrates

c) Excessive uptake of protein

d) Cocaine abuse, folic acid deficiency

24. Clinical features of abruptio placentae are:

a) Painful, revealed, concealed or mixed, dark-colored bleeding

b) Painless, only revealed, dark-colored vaginal bleeding

c) Painful, only concealed, dark-colored bleeding

d) Painless, only concealed, dark-colored bleeding

25. All the investigations are done in abruptio placentae, except :

a) Hemoglobin and hematocrit estimation.

b) Coagulation profile

c) ABO and Rh grouping

d) Beta-HCG

26. Separation of placenta is affected by:

a) External version

b) Premature rupture of membranes

c) Traumatic injuries

d) All of the above

27.  In placenta previa, vaginal bleeding is:

a) Concealed

b) Revealed

c) Mixed

d) No bleeding

28. Complication of abruptio placentae is/are:

a) Uterine prolapse

b) Genital prolapse

c) Cervical incompetency

d) Hemorrhagic shock

29. Vaginal examination is contraindicated in :

a) Placenta previa

b) Placenta previa or undiagnosed APH

c) Diagnosed APH

d) Diagnosed placenta previa.

30. Clinical manifestation of abruptio placentae is:

a) Uterine activity

b) Intense abdominal pain

c) Headache

d) Cramping

Section C

Practice Assessment Checklist to Assess the Expressed Practices Regarding Obstetrical Emergencies Among Staff Nurses

Sr. No.

Statements

Yes

No

1.

Rolling of eyes to one side with the fixed stare is the first objective sign of a convulsion in a client with eclampsia.

2.

Excessive weight gain is the earliest sign of pregnancy-induced hypertension.

3.

The prognostic point of view suggests a diastolic rise of blood pressure is more than the systolic rise.

4.

Diet rich in carbohydrates may reduce the risk of PIH.

5.

Half an hourly pulse, respiration and blood pressure should be monitored in eclamptic client.

6.

In eclamptic client, per day fluid intake should exceed the previous 24 hours urinary output + 1000ml.

7.

Therapeutic level of serum magnesium is 4-7 Meq/l.

8.

In premonitory stage, mouth gag is placed to prevent tongue bite.

9.

During airway clearance, the client’s head is not to turned to one side and the pillow is not taken off.

10.

Loading dose of Inj. Magnesium sulfate is given 4gm I/V over 3-5 minutes.

11.

Oxygen may be administered through face mask at 8-10 L/min to prevent or minimize fetal hypoxia.

12.

In placenta previa, the perineal pads should be weighed to estimate the blood loss i.e. 1g = 1 ml.

13.

During management of obstetrical emergencies intake output should be maintained every hourly.

14.

Separation of placenta is not affected by trauma including vaginal examination and coital act.

15.

Repeat USG is done at 34 weeks of gestation to confirm the low-lying placenta previa.

16.

Coffee color vomiting is not due to subcapsular hemorrhage in pre-eclampsia.

17.

Calcium supplements increase the risk of gestational hypertension.

18.

 Patient is advised to lie in the left lateral position to avoid supine hypotension.

19.

Routine intake of folic acid reduces the chances of abruptio placentae.

20.

Eclamptic client is not at risk for any complications in subsequent pregnancy.

Answer Keys

Section B

1. A

1. A                                                                             26. D                                                                      

2. A                                                                            27. B

3. A                                                                            28. D

4. D                                                                            30.B

5. D                                                                           

6. B

7. D

8. B

9. C

10. A

11. C

12. A

13. B

14. C

15. A

16. C

17. A

18. B

19. A

20. B

21. D

22. A

23. D

24. D

25. C

Section C

1. Yes

2. Yes

3. Yes

4. No

5. Yes

6. No

7. Yes

8. Yes

9. Yes

10. Yes

11. Yes

12. Yes

13. Yes

14. No

15. Yes

16. No

17. No

18. Yes

19. Yes

20. No
